# Advancing urban ethnopharmacology: a modern concept of sustainability, conservation and cross-cultural adaptations of medicinal plant lore in the urban environment

**DOI:** 10.1093/conphys/coab073

**Published:** 2021-09-16

**Authors:** Tusheema Dutta, Uttpal Anand, Suchismita Chatterjee Saha, Abhijit Bhagwan Mane, Dorairaj Arvind Prasanth, Ramesh Kandimalla, Jarosław Proćków, Abhijit Dey

**Affiliations:** 1 Ethnopharmacology and Natural Product Research Laboratory, Department of Life Sciences, Presidency University, 86/1 College Street, Kolkata, 700073, West Bengal, India; 2 Department of Life Sciences, Ben-Gurion University of the Negev, Beer-Sheva, 84105, Israel; 3 Department of Zoology, Nabadwip Vidyasagar College (Affiliated to the University of Kalyani), Nabadwip, West Bengal, 741302, India; 4 Department of Zoology, Dr. Patangrao Kadam Mahavidyalaya, Sangli, (Affiliated to Shivaji University of Kolhapur), Maharashtra, 416308, India; 5 Department of Microbiology, School of Biosciences, Periyar University, Salem, 636011, Tamilnadu, India; 6 CSIR-Indian Institute of Chemical Technology, Uppal Road, Tarnaka, Hyderabad, 500007, Telangana, India; 7 Department of Biochemistry, Kakatiya Medical College, Warangal, 506007, Telangana, India; 8 Department of Plant Biology, Institute of Environmental Biology, Wrocław University of Environmental and Life Sciences, Kożuchowska 5b, 51-631 Wrocław, Poland

**Keywords:** Conservationenvironmentmedicinal plantsacred grovessustainabilityUrban ethnopharmacology

## Abstract

The discipline ‘urban ethnopharmacology’ emerged as a collection of traditional knowledge, ancient civilizations, history and folklore being circulated since generations, usage of botanical products, palaeobotany and agronomy. Non-traditional botanical knowledge increases the availability of healthcare and other essential products to the underprivileged masses. Intercultural medicine essentially involves ‘practices in healthcare that bridge indigenous medicine and western medicine, where both are considered as complementary’. A unique aspect of urban ethnopharmacology is its pluricultural character. Plant medicine blossomed due to intercultural interactions and has its roots in major anthropological events of the past. Unani medicine was developed by Khalif Harun Al Rashid and Khalif Al Mansur by translating Greek and Sanskrit works. Similarly, Indo-Aryan migration led to the development of Vedic culture, which product is Ayurveda. Greek medicine reached its summit when it travelled to Egypt. In the past few decades, ethnobotanical field studies proliferated, especially in the developed countries to cope with the increasing demands of population expansion. At the same time, sacred groves continued to be an important method of conservation across several cultures even in the urban aspect. Lack of scientific research, validating the efficiency, messy applications, biopiracy and slower results are the main constrains to limit its acceptability. Access to resources and benefit sharing may be considered as a potential solution. Indigenous communities can copyright their traditional formulations and then can collaborate with companies, who have to provide the original inventors with a fair share of the profits since a significant portion of the health economy is generated by herbal medicine. Search string included the terms ‘Urban’ + ‘Ethnopharmacology’, which was searched in Google Scholar to retrieve the relevant literature. The present review aims to critically analyse the global concept of urban ethnopharmacology with the inherent plurality of the cross-cultural adaptations of medicinal plant use by urban people across the world.

## Teaser

Urban ethnopharmacology is rapidly advancing and gaining significant attention throughout the world. In this review, we have presented the potential of urban ethnopharmacology and medicinal plants from the anthropologist and religious viewpoints. Additionally, the emergence and the present status of urban ethnopharmacology throughout the globe have been critically reviewed.

## Introduction

A confluence of anthropology, history, flow of traditional folklore through generations, palaeobotany, ethnomedicine and agronomy gave birth to the discipline of urban ethnopharmacology. Plant-based medicines have been popular prior to the arrival of modern synthetic drugs. The study of plants and plant-based products used by city and town dwellers is known as urban ethnopharmacology, as emphasized by the prefix ‘urban’. In a nutshell, the complicated relationships between people and plants in towns and cities form the basis of urban ethnopharmacology (Albuquerque *et al.*, 2014; Hurrell and Pochettino, 2014; Anand *et al.*, 2019; Mohammed *et al.*, 2021; Datta *et al.*, 2021). Urban botanical knowledge encompasses the consumption criteria of selection and production of plant products as well as the role of such knowledge in conservation (Hurrell and Pochettino, 2014). Lately, urban ethnopharmacology has been given crucial recognition, because a number of these traditionally used species are now vulnerable or critically endangered. International migrations lead to the expansion of traditional knowledge. When migrants settle in new cities or towns, they continue using their traditional remedies despite the availability of allopathic medicines and other conventional drugs. These dynamics of change upon being introduced from other communities into the existing ethnopharmacological implementations are explored and scrutinized in urban ethnopharmacology (Ceuterick *et al.*, 2008; Pieroni and Privitera, 2014).

The fact that the literature available in this field is limited as this knowledge has traditionally been transmitted orally from one generation to the next and the only literature available is in the form of vernacular trivial ethnographic publications is a major constraint on the inclusion of traditional medicine in public health care practices (Petkeviciute *et al.*, 2010). Some researchers, like Jacques, Barrau and Villamar, even consider ethnopharmacology as a section of anthropology, completely denying its existence in Botany (Albuquerque *et al.*, 2014). Another limitation might be the study of only the economically important plants of particular regions by earlier ethnobotanists, vehemently omitting the plants that were found in that region but were utilized in other regions, a practice condemned by (Kroeber, 1920, Hurrell, 2014). An important feature of non-traditional botanical knowledge (Hurrell and Pochettino, 2014) is the availability of healthcare and other essential products to the underprivileged masses (Ocvirk *et al.*, 2013; Vandebroek, 2013).

Intercultural medicine essentially involves ‘practices in healthcare that bridge indigenous medicine and western medicine, where both are considered as complementary’ (Mignone *et al.*, 2007; Vandebroek, 2013). Cities are assumed to be systems where society and nature form a fused heterogeneous ecosystem within which synthetic and organic components are interrelated (Almada, 2010; Ladio and Albuquerque, 2014). Since ecology as a discipline flourished beforehand, it is important to analyse whether the order of anthropological usage of herbs matches with the predictions made by existing ecological theories and concepts, which can only be done if the ethnobotanical concepts and theories of various cultures are systematically recognized and developed into hypotheses that can be validated experimentally (Gaoue *et al.*, 2017). One of the shortcuts is the usage of hybridization, where traditional medicine is reconstructed and integrated in different ways to form a product more compatible for urban markets (Ladio and Albuquerque, 2014). On the other hand, an important question arises: ‘Who will get the commercial benefits, these individual migrant groups or researchers, where the concept of intellectual property rights (IPR) is to be considered?’ (McGonigle, 2016). Some ethnobotanists consider children to be important preservers and informers of botanical knowledge, passing knowledge to each other, being remnants of practices no longer present in adults and trying newer species while playing in nature and interacting with the environment (Łuczaj and Nieroda, 2011), whereas another set of researchers believe the women as reservoirs of traditional botanical knowledge (TBK) (Voeks, 2007; Katiyar *et al.*, 2012; Wayland and Walker, 2014). Urban ethnobotany, introduced lately as a terminology in modern ethnobotany, truly depicts the ‘pluricultural contexts in the urban agglomerations’ with its ostensible inclination to the traditional field work with a need for sustainable conservation of urban folklore. Earlier, only few works have been carried out on modern urban ethnobotany in Latin American and European contexts. The present review aims to comprehensively enumerate the practices of urban ethnopharmacology across the world with notes on sustainable utilization of medicinal plants and the inherent plurality of the cross-cultural adaptations of medicinal plant use by the urban people. This review, in true sense, is a piece of multidisciplinary endeavour where history, social studies, botany and ethical issues present their own shares of theoretical and methodological contributions.

## Methodology

To retrieve the relevant records, a literature review of multiple disciplines from 1922 to 2019 was performed. The search string included the term ‘Urban’ + ‘Ethnopharmacology’, which was searched in Google Scholar, PubMed-NCBI, SpringerLink, Nature Publishing Group and other scientific databases. A detailed analysis of the articles presented in the references section was performed. Species names were checked against The Plant List 1.1 (2013), and family names follow the Angiosperm Phylogeny Group IV (Chase *et al.*, 2016). All figures were constructed with the help of Microsoft Word, Autodesk or Sketchbook or were hand drawn. All tables and graphs were constructed by Microsoft Word and PowerPoint.

## Through the eye of an anthropologist: the cross-cultural nature of urban ethnopharmacology

**Figure 1:**
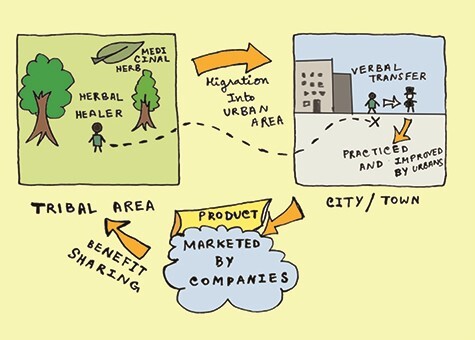
The spread and development of urban ethnopharmacology.

A unique aspect of urban ethnopharmacology is its pluricultural character. Pluriculturalism is a concept where individual identities result from participating in different cultures (Martínez *et al.*, 2006). Multiculturalism is a theory where a community comprises of several ethnic groups, which may or may not have interactions. Pluriculturalism is based on the different interactions between these ethnic groups and new knowledge evolving from this exchange of values, traditions and resources. Multiculturalism forces uniformity and consistency and pluriculturalism allows for fluidity. Rural and small ethnic groups, who remain isolated from each other in their original habitats, tend to interact when they settle in urban areas (Fig. 1).

These migrations lead to pluralism or the doctrine of multiplicity (Schneider, 2007), which is the co-existence of ideas and principles. Conflict of ideas creates more ideas and hence confluence and conflict of knowledge creates more knowledge. Therefore, contrasting ethnopharmacology leads to the advancement of ethnopharmacology and drug delivery. Historical events sometimes influence the propagation of ethnopharmacology as well. For example, an increased trend of using medicinal plants was seen in Europe during the aftermath of World War II as a result of the scarcity of resources (Pardo-de-Santayana *et al.*, 2015). Unani medicine was developed by Khalif Harun Al Rashid and Khalif Al Mansur by translating Greek and Sanskrit works (Dahanukar and Thatte, 1997). Similarly, Indo-Aryan migration led to the development of Vedic culture resulted in Ayurveda (Childe, 1996). Greek rational medicine reached its summit when it travelled to Egypt (Longrigg, 2013). Garlic [*Allium sativum* L. (Amaryllidaceae)], a medicinal plant widely used in India for cardio-vascular disorders and lowering cholesterol, was found in the tombs of Egyptian pharaohs and Greek temples, used against respiratory disorders in Rome, flu in America, plague in Europe and for digestion in China and Japan. (Gebreyohannes and Gebreyohannes, 2013; Alqethami *et al.*, 2017). The medicinal use of Neem [*Azadirachta indica* A. Juss. (Meliaceae)] started in China when Bhogar Sidhdhar travelled from India to teach in China (Kumar and Navaratnam, 2013). These historical studies clearly show that plant medicine blossomed due to intercultural interactions and has its roots in major anthropological events of the past. Archaeological evidence even showed that botanical products and cereal foods were consumed by prehistoric men (Day, 2013; Valamoti*et al.*, 2019) and selective consumption of plants by monkeys were interestingly similar to the ones used by humans (Weiner, 1980) demonstrating the origins of ethnopharmacology even beyond human origins. The synthesis of these compounds by plant species were itself a result of interactions with microbes, pests and herbivores (Bhattacharya, 2014).

## Urban ethnopharmacology: emergence and present status throughout the globe

In the past few decades, the ethnobotanical field studies proliferated, especially in the developed countries to cope with the increasing demands of population expansion (Pieroni and Privitera, 2014). All studies included selection of a specific site of study, its detailed description including its geography, geology, ethnic background, ecology, edaphology, pedology, history, etc., description of sampling methods, proper interviews conducted with the participants, scientific identification of the species involved and data analysis by statistical methods including comparison with previous data (Cunningham, 2001; de Albuquerque and Hurrell, 2010; de Medeiros *et al.*, 2013; Akbulut and Bayramoglu, 2014; Albuquerque *et al.*, 2014; Conde *et al.*, 2014; Pieroni and Privitera, 2014; Akbulut, 2015; Taylor and Lovell, 2015) or with the help of bioinformatics (Torre *et al.*, 2012; Quave *et al.*, 2012; Lagunin *et al.*, 2014) The questions addressed are as follows:

- How TBK accounts for sustainability and selection?
- What are the roles of a specific species or genus in a society or community?
- Evaluation of this knowledge for satisfying community needs.

These studies prevailed across the globe. A brief account of the ethnobotanical literature has been given in the Supplementary Table 1, where the main families and species uses have been described.

### Urban ethnopharmacology in north and south American countries

A large number of South American literatures, especially from Brazil, were found regarding the use of plants as alternative medicine in urban areas. Extensive studies have been done in a number of South American cities like Petrópolis, Nova Friburgo, Rio Claro, Abaetetuba, etc. (Hurrell *et al.*, 2015). This shows the importance and preservation of traditional knowledge among the urban population. However, in spite of these practices, ethnobotanical knowledge was found to be inversely proportional to financial status and education in Brazil (Arenas *et al.*, 2013) drawing attention to its conservation and widespread awareness. North America showed contrasting results. Limited jobs and lack of funding is a considerable limitation (Barkaoui *et al.*, 2017), and hence TBK mainly was under practice in some parts of Mexico (Saynes-Vásquez *et al.*, 2016; Lara Reimers *et al.*, 2019). Occasionally, children from privileged families having private insurance in the USA were seen using complementary and alternative medicine (CAM) as a supplement to conventional medicine (Barnes *et al.*, 2008).

In Brazil, De Melo *et al.* studied various medicinal plants having anticancer properties. A total number of 84 plants were listed, out of which *Aloe vera* (L.) Burm. f. (Asphodelaceae), *Euphorbia tirucalli* L. (Euphorbiaceae) and *Handroanthus impetiginosus* (Mart. ex DC.) Mattos [= *Tabebuia impetiginosa* (Mart. ex DC.) Standl., Bignoniaceae] with the molecules of silibinin, β-lapachone, plumbagin and capsaicin were studied both *in vivo* and *in vitro* (De Melo *et al.*, 2011). In the open fairs of Petrópolis and Nova Friburgo, Rio de Janeiro, 94 species of medicinal plants were reported, with the families of Asteraceae (26 species) and Lamiaceae (10 species) being the most frequently represented. Common species included *Ageratum conyzoides* (L.) L. (Asteraceae), *Dasyanthina serrata* (Less.) H.Rob. (= *Vernonia serrata* Less., Asteraceae), *Baccharis dracunculifolia* DC. (Asteraceae), *Mentha pulegium* L. (Lamiaceae), *Rosmarinus officinalis* L. (Lamiaceae), etc. (Eichemberg *et al.*, 2009). In a review article by Abreu *et al.*, a total of 717 medicinal plant species were identified, out of which *R. officinalis*, *Ruta graveolens* L. (Rutaceae), *Aloe arborescens* Mill. (Asphodelaceae), *Bidens pilosa* L. (Asteraceae) and *Plectranthus barbatus* Andrews (Lamiaceae) were the most important documented plants in Brazilian ethnobotanical literature (Abreu *et al.*, 2015; De Melo *et al.*, 2011). The urban old gardens of Rio Claro house 93 species of medicinal plants with Rutaceae, Euphorbiaceae, Araceae, Asteraceae and Solanaceae as the most representative families. Notable species included *Tanacetum parthenium* (L.) Sch.Bip. [= *Chrysanthemum parthenium* (L.) Bernh., Asteraceae], *Zinnia elegans* L. (Asteraceae), *Lactuca sativa* L. (Asteraceae), *Begonia bowerae* Ziesenh. (= *B. boveri* Ziesenh., Begoniaceae) etc. (Eichemberg *et al.*, 2009; Amorozo, 2002). In the home gardens of Abaetetuba, a city in Pará state, Brazil, 124 species were identified, 17.6% of which was used for curing infectious and parasitic diseases. *Hemigraphis colorata* W. Bull (Acanthaceae) was used to treat haemorrhoids and ear infections; *Justicia pectoralis* Jacq. (Acanthaceae) for uterine infections; *Justicia secunda* Vahl for gastric ailments; *Sambucus nigra* L. (Adoxaceae) for flu measles, chickenpox, wounds and cough; *Alternanthera brasiliana* (L.) Kuntze (Amaranthaceae) for urinary tract infections; *Dysphania ambrosioides* (L.) Mosyakin & Clemants (Amaranthaceae) for asthma; *Mangifera indica* L. (Anacardiaceae) for diarrhea; *Annona muricata* L. (Annonaceae) for obesity and diabetes; and *Eryngium foetidum* L. (Apiaceae) for intestinal worms (Palheta *et al.*, 2017; WinklerPrins and Oliveira, 2010). A total number of 129 medicinal plants were found to be used in La Paz and El Alto cities in Bolivia, including the following: *Pimpinella anisum* L. (Apiaceae) for the treatment of diarrhea and stomach ache; *Baccharis genistelloides* (Lam.) Pers. (Asteraceae) for diabetes; *Mutisia acuminata* Ruiz & Pav. (Asteraceae) for dizziness, headache and kidney ailments; and *Xanthium spinosum* L. (Asteraceae) for measles and chicken pox etc. (Macía *et al.*, 2005).

**Figure 2:**
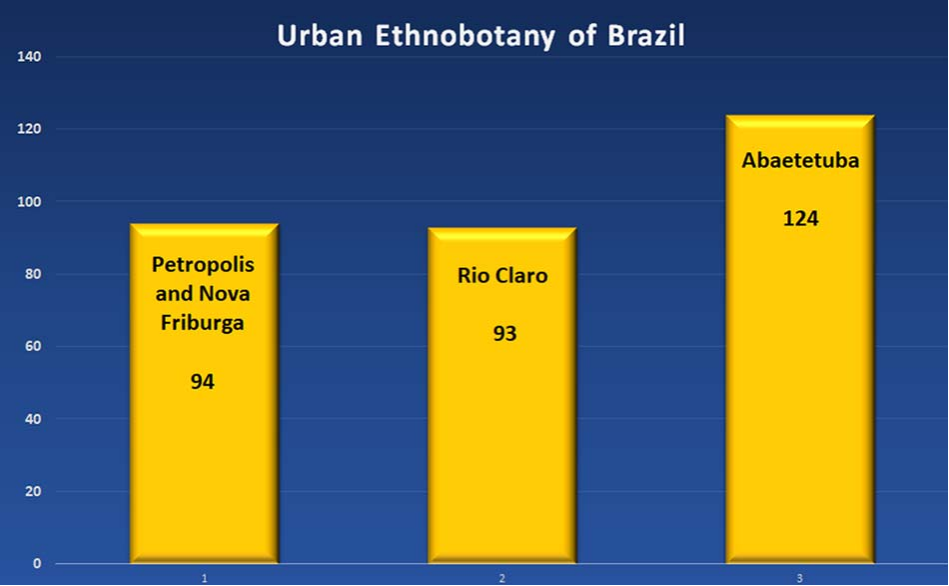
Graphical ethnobotanical data from four Brazilian urban areas. Y-axis, number of medicinal plant species in urban home gardens.

Fifty species of edible and medicinal plants were found in a study conducted at markets of Bolivian immigrants in Buenos Aires, Argentina. *Coriandrum sativum* L. (Apiaceae), *Baccharis articulata* (Lam.) Pers. (Asteraceae), *Opuntia ficus-indica* (L.) Mill. (Cactaceae), *Lepidium meyenii* Walp. (Brassicaceae), etc., were some of the notable plants found (Arenas *et al.*, 2013; Pochettino *et al.*, 2012). A graphical representation of these information is presented in Fig. 2.

In Mexico, herbal medicine is used by more than 90% of the population (Popoca *et al.*, 1998). *Fucus vesiculosus* L. (Fucaceae), *Citrus* × *aurantium* L. (Rutaceae), *Citrus limon* (L.) Osbeck (Rutaceae), *Hibiscus sabdariffa* L. (Malvaceae), etc., were used against obesity (Arenas *et al.*, 2013). A total of 181 plant species having potent antitumor effects were identified (Alonso-Castro *et al.*, 2011), including *Justicia spicigera* Schltdl. (Acanthaceae), *Agave salmiana* Otto ex Salm-Dyck (Asparagaceae) (Popoca *et al.*, 1998), *B. pilosa* L. (Asteraceae) (Nieto *et al.*, 2008), *Dendropanax arboreus* (L.) Decne. & Planch. (Araliaceae) (Hernández, 1959), *Aristolochia brevipes* Benth. (Aristolochiaceae), etc. Other medicinal plants are *Agave vilmoriniana* A. Berger (Asparagaceae), *D. ambrosioides* (L.) Mosyakin & Clemants (= *Chenopodium ambrosiodes* L., Amaranthaceae), *Cosmos pringlei* B.L. Rob. & Fernald (Asteraceae), *Jatropha* sp. (Euphorbiaceae), *Zornia* sp. (Fabaceae), etc. (Bye, 1986). However, with the advent of modernization and better quality of living, erosion of this knowledge is prevalent (Bensoussan *et al.*, 1998).

Ethnopharmacology as a discipline is still under development in the USA due to limited job opportunities (Barkaoui *et al.*, 2017). Brazilian Candomblé and Santería immigrants in New York use medicinal plants of Brazilian, Latino and West African origins like *Allium cepa* L. (Amaryllidaceae), *A. vera* (Asphodelaceae), *Amaranthus hybridus* L. (Amaranthaceae), *Ambrosia artemisiifolia* L. (Asteraceae), *A. indica* A. Juss. (Meliaceae), *D. ambrosioides* (L.) Mosyakin & Clemants (= *Chenopodium ambrosiodes* L., Amaranthaceae), *Cocos nucifera* L. (Arecaceae), *Cinnamomum cassia* (L.) J. Presl (= *C. aromaticum* Nees, Lauraceae), *Dracaena fragrans* (L.) Ker Gawl. (Asparagaceae), *Mentha* sp. (Lamiaceae), *Mimosa pudica* L. (Fabaceae), *Nicotiana tabacum* L. (Solanaceae), etc. (Fonseca and Balick, 2018). Latino healers use *Achillea millefolium* L. (Asteraceae), *Daucus carota* L. (Apiaceae), *Eucalyptus* sp. (Myrtaceae), *R. officinalis* L. (Lamiaceae), etc., for uterine fibrosis; *Plantago major* L. (Plantaginaceae), *Ruta chalepensis* L. (Rutaceae), *P. anisum* L. (Apiaceae)*, O. ficus-indica* (L.) Mill. (Cactaceae) and *Tilia mandshurica* Rupr. & Maxim. (Malvaceae) for menorrhagia; and *Citrus* sp. (Rutaceae) and *Zingiber officinale* Roscoe (Zingiberaceae) for hot flushes (Augustino and Gillah, 2005). Chinese and Taiwanese immigrants in the metro-Atlanta area use *A. cepa*, *A. sativum* (both Amaryllidaceae), *Cucurbita* sp. (Cucurbitaceae), *Ginkgo biloba* L. (Ginkgoaceae), *Bambusa oldhamii* Munro (Poaceae)*, Lycium chinense* Mill. (Solanaceae), *Z. officinale* (Zingiberaceae), etc., for the preparation of medicinal foods (Jiang and Quave, 2013). Knowledge about local plants was also found in the non-native students of Arizona (O’Brien, 2010). American Indians utilize *Eupatorium perfoliatum* L. (bonest, Asteraceae), *Podophyllum peltatum* L. (mayapple, Berberidaceae), *Panax quinquefolius* L. (ginseng, Araliaceae), etc., whereas in Honduras medicinal plants are widely used by Miskitos, Sumus, Pech and Lencas, Pipiles of El Salvador and Talamancas of Costa Rica (Hoareau and DaSilva, 1999).

In Canada, 400 species of medicinal plants are used by the native people living in Montreal, Quebec, and Ontario, including *Populus balsamifera* L. (Salicaceae), *Thuja occidentalis* L. (Cupressaceae), *Geranium maculatum* L. (Geraniaceae), etc. (Arnason *et al.*, 1981), although TBK has suffered erosion in recent years (Turner and Turner, 2008).

### Urban ethnopharmacology in Europe

**Figure 3:**
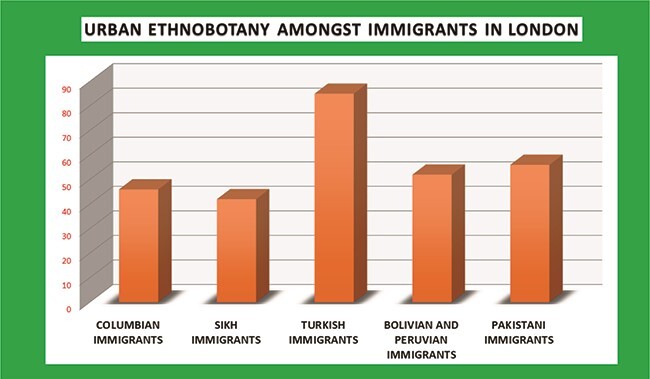
Graph of ethnobotanical data of London. Y-axis, number of medicinal plant species used by different groups of immigrants.

In Europe, increased dependence on natural remedies can be seen in recent years (Coady and Boylan, 2014; Pardo-de-Santayana *et al.*, 2015; Petrakou *et al.*, 2020). Use of natural products escalated after the Second World War, due to considerable financial and resource losses (Pardo-de-Santayana *et al.*, 2015). Interestingly, a number of literatures are recorded from London, England, where five types of immigrants have a rich knowledge of herbal medicine (Fig. 3**)**. This is way different from the information found from USA, another developed first world nation. The *Thymus vulgaris* L. (Lamiaceae), *S. nigra* L. (Adoxaceae)*, Santolina chamaecyparissus* L. (Asteraceae), *A. cepa*, etc., were used in Spain; *Geranium purpureum* Vill. (Geraniaceae), *Phlomis purpurea* L. (Lamiaceae), *M. pulegium* L. (Lamiaceae), *Juglans regia* L. (Juglandaceae), etc, in Portugal; and *Chelidonium majus* L. (Papaveraceae), *Crataegus monogyna* Jacq. (Rosaceae), *Chamaemelum nobile* (L.) All. (Asteraceae), *Foeniculum vulgare* Mill. (Apiaceae), *Malva sylvestris* L. (Malvaceae), *M. pulegium, Paronychia argentea* Lam. (Caryophyllaceae), *S. chamaecyparissus, R. officinalis* and *S. nigra* were used in Greece and Turkey (Quave *et al.*, 2012; Akaydin *et al.*, 2013, Kilic and Bagci, 2013; Yesilada, 2013; Akbulut and Bayramoglu, 2014). Home remedies were used to treat common disorders such as catarrh, pneumonia, fever, diarrhoea, stomach and intestinal disorders, high blood pressure, wounds, bruises or muscular pain (Carvalho, 2010; Quave, and Pieroni, 2015). Colombian communities in London use 46 plant species as herbal medicine like *M. indica* (Anacardiaceae) and *Anethum graveolens* L. (Apiaceae) for stomach ailments, *Petroselinum crispum* (Mill.) Fuss (Apiaceae) for epilepsy, *Erythroxylum coca* Lam. (Erythroxylaceae) for dental problems, etc. (Ceuterick *et al.*, 2008). Forty two species were found to be used by the Sikh groups in London with *A. cepa, A. sativum, Capsicum annuum* L. (= *C. frutescens* L., Solanaceae), *Cinnamomum verum* J. Presl (Lauraceae)*, C. limon, F. vulgare, Elettaria cardamomum* (L.) Maton and *Z. officinale* Roscoe (both latter Zingiberaceae) serving as the most common species (Sandhu and Heinrich, 2005). Turkish speaking Cypriots treat 13 ailments with the help of 85 different plants like *Olea europaea* L. (Oleaceae), *C. limon, Eucalyptus camaldulensis* Dehnh. (Myrtaceae), *S. nigra, Urtica urens* L. (Urticaceae), *A. cepa, A. sativum, C. verum* J.Presl (= *C. zeylanicum* Blume, Lauraceae), *M. sylvestris* L.*/parviflora* L. (Malvaceae), *Origanum syriacum* L. (Lamiaceae), *Saxifraga hederacea* L. (Saxifragaceae) and *Tilia cordata* Mill. (Malvaceae) (Yöney *et al.*, 2010). Bolivian and Peruvian migrants use *Matricaria chamomilla* L. (= *M. recutita* L., Asteraceae), *Carica papaya* L. (Caricaceae), *Gnaphalium versatile* Rusby (Asteraceae), *Eucalyptus globulus* Labill. (Myrtaceae), *P. major, Chenopodium quinoa* Willd. (Amaranthaceae), *Morinda citrifolia* L. (Rubiaceae), etc. (Ceuterick *et al.*, 2011). Pakistani migrants use 56 plant-based Unani medicines for different ailments in Bradford (Pieroni *et al.*, 2008). Senegalese community living in Turin, Italy, uses *Acacia nilotica* (L.) Delile (Fabaceae) for toothache, *Adansonia digitata* L. (Malvaceae) for diarrhoea, *C. limon* for malaria, *Euphorbia balsamifera* Aiton (Euphorbiaceae) for wounds and *Syzygium aromaticum* (L.) Merr. & L.M.Perry (= *Eugenia caryophyllata* Thunb., Myrtaceae) for eye problems (Fassina *et al.*, 2002). Venetian migrants from Romania have 135 plant-based food and medicinal preparations including the species of *A. millefolium, Amaranthus retroflexus* L. (Amaranthaceae), *Artemisia abrotanum* L. (Asteraceae), *Calendula officinalis* L. (Asteraceae), *Iris* × *germanica* L. (Iridaceae), *N. tabacum*, *Ocimum basilicum* L. (Lamiaceae), etc. (Borza, 1968; Pieroni *et al.*, 2012). *C. officinalis, Valeriana officinalis* L. (Caprifoliaceae), *Hypericum perforatum* L. (Hypericaceae), *Artemisia absinthium* L. (Asteraceae), *A. millefolium, Acorus calamus* L. (Acoraceae) and *Aesculus hippocastanum* L. (Sapindaceae) are the most widely used plants in urban Samogitia region, Lithuania (Petkeviciute *et al.*, 2010). In French Guiana, 226 medicinal and cosmetic plants are used by the urban youth, including *Aristolochia trilobata* L. (Aristolochiaceae), *Carapa guianensis* Aubl. (Meliaceae), *Cannabis sativa* L. (Cannabaceae), *Orthosiphon aristatus* (Blume) Miq. (Lamiaceae), etc. (Tareau *et al.*, 2017). Urban populations in Paramaribo, Suriname utilize 144 medicinal plants with *Gossypium barbadense* L. (Malvaceae), *Phyllanthus amarus* Schumach. & Thonn. (Phyllanthaceae) and *Quassia amara* L. (Simaroubaceae) being the most frequently mentioned species (van Andel and Carvalheiro, 2013).

### Urban ethnopharmacology in Africa

Africa is a continent with a rich biodiversity and miscellaneous ethnic groups. Each ethnic group has their own knowledge and medicinal practices. As the regions developed and the urban and rural divisions became blurred, these traditions seeped into the urban populations (Oreagba *et al.*, 2011). Several of these practices are now prevalent in cities (Amira and Okubadejo, 2007; Hughes *et al.*, 2015). Bush doctors of Western Cape in South Africa treat more than 30 illnesses including 12 gastrointestinal symptoms, urogenital infections, skin problems and cardiovascular diseases with the help of 181 plant species like *Acacia dealbata* Link (Fabaceae), *Agathosma crenulata* (L.) Pillans (Rutaceae), *Aloe ferox* Mill. (Asphodelaceae), *Asparagus* sp. (Asparagaceae), *Crossyne guttata* (L.) D. Müll.-Doblies & U.Müll.-Doblies (Amaryllidaceae), *Dioscorea sylvatica* Eckl. (Dioscoreaceae), *Gnidia capitata* L.f. (Thymelaeaceae), *Lavandula angustifolia* Mill. (Lamiaceae), *Pelargonium triste* (L.) L’Hér. (Geraniaceae), *R. graveolens*, *Salvia africana-lutea* L. (Lamiaceae), *Zanthoxylum capense* (Thunb.) Harv. (Rutaceae), etc. (Philander, 2011; Maroyi and Mosina, 2014). In Kenya, 13 plant species—*Aloe deserti* A. Berger (Asphodelaceae), *Launaea cornuta* (Hochst. ex Oliv. & Hiern) C. Jeffrey (Asteraceae), *O. basilicum* L. (Lamiaceae), *Vepris simplicifolia* (Engl.) Mziray [= *Teclea simplicifolia* (Engl.) I. Verd., Rutaceae], *Gerrardanthus lobatus* (Cogn.) C. Jeffrey (Cucurbitaceae), *Grewia hexamita* Burret (Malvaceae), *Canthium glaucum* Hiern (Rubiaceae), *A. hybridus* L. (Amaranthaceae), *Combretum padoides* Engl. & Diels (Combretaceae), *Senecio pinifolius* Dusén (Asteraceae), *Ocimum gratissimum* L. (= *O. suave* Willd., Lamiaceae), *Aloe macrosiphon* Baker (Asphodelaceae) and *Landolphia buchananii* (Hallier f.) Stapf (Apocynaceae)—are used in the treatment of malaria (Njoroge and Kibunga, 2007, Nguta *et al.*, 2010). In urban districts of Tanzania, some of the notable herbal medicines used (Mhame, 2000) are as follows: *Abrus precatorius* L*.* (Fabaceae) for treating viral and bacterial infections, stomach ache, eye ache, haemorrhoids; *Acacia polyacantha* Willd. (Fabaceae) for labour pain and infertility; *A. digitata* L. (Malvaceae) for tuberculosis; *Albizia harveyi* E. Fourn. (Fabaceae) for snake bites; and *Catharanthus roseus* (L.) G.Don (Apocynaceae) for hypertension and diabetes mellitus. In Moroccan urban areas, cutaneous infections were treated by *A. cepa* L., *Lawsonia inermis* L. (Lythraceae), *Lepidium sativum* L. (Brassicaceae), *Artemisia herba-alba* Asso (Asteraceae), *Carlina gummifera* (L.) Less. (= *Atractylis gummifera* Salzm. ex L., Asteraceae), *C. limon* (L.) Osbeck (Rutaceae), *Marrubium vulgare* L., *Salvia verbenaca* L. (both latter Lamiaceae), *Quercus infectoria* G. Olivier (Fagaceae), *Solanum lycopersicum* L. (Solanaceae), *R. officinalis* L. (= *Salvia rosmarinus* Schleid., Lamiaceae), *A. sativum* L. (Amaryllidaceae), *Argania spinosa* (L.) Skeels (Sapotaceae), *Carpobrotus edulis* (L.) N.E.Br. (Aizoaceae), *O. europaea* L. (Oleaceae), *Ziziphus jujuba* Mill. (= *Z. vulgaris* Lam., Rhamnaceae), *Tetraclinis articulata* (Vahl) Mast. (Cupressaceae) and *Juniperus oxycedrus* L. (Cupressaceae), whereas *A. sativum*, *Salvia officinalis* L., *Marrubium vulgare* L. and *Lavandula dentata* L. (last three belong to Lamiaceae) were used as anti-diabetic agents (Ouhaddou *et al.*, 2014; Makbli *et al.*, 2016; Barkaoui *et al.*, 2017).

### Urban ethnopharmacology in Asia

Asia is well known for its rich cultural heritage and biodiversity. As a result, a large number of publications were recorded on the modern-day usage of botanical products in urban regions. The literature was mainly from Southern Asia, having both developed and developing nations (Astutik *et al.*, 2019). China, India, Pakistan, Bangladesh and Indonesia had the most publications. Use of plants has been prevalent in Chinese traditional medicine (Tu, 2011; Tang and Eisenbrand, 2013). *Ephedra sinica* Stapf (Ephedraceae) was found to be effective against constipation, *Artemisia annua* L. (Asteraceae) could kill Quinine resistant *Plasmodium* sp. and *Coix lacryma-jobi* L. (Poaceae) had potent anti-tumour effects (Normile, 2003). A total of 20 traditional Chinese medicine was useful against irritable bowel syndrome in Sydney, Australia. The species included *Codonopsis pilosula* (Franch.) Nannf (Campanulaceae), *Z. officinale* Roscoe (Zingiberaceae), etc. (Bensoussan, 1998). Traditional Indian medicine or Ayurveda has similarities with traditional Chinese medicine (Yuan *et al.*, 2016). Species commonly used in Ayurveda are as follows: *Sesbania grandiflora* (L.) Pers. (Fabaceae), *Phyllanthus emblica* L. (Phyllanthaceae), *Terminalia arjuna* (Roxb. ex DC.) Wight & Arn. (Combretaceae), *Saraca asoca* (Roxb.) Willd. (Fabaceae), *Withania somnifera* (L.) Dunal (Solanaceae), *Ficus religiosa* L. (Moraceae), *Santalum album* L. (Santalaceae), *Terminalia chebula* Retz. (Combretaceae), *Piper betle* L. (Piperaceae), etc. (Sharma *et al.*, 2005, Parasuraman *et al.*, 2014). *Commiphora* sp. (Burseraceae) for controlling cholesterol, *Picrorhiza* sp. (Plantaginaceae) for liver problems, *Bacopa* sp. (Plantaginaceae) for improving memory, *Curcuma* sp. (Zingiberaceae) as an anti-allergic medicine and *Asclepias* sp. (Apocynaceae) against cardiac disorders are some of the recent developments in the commercial use of ancient herbal knowledge (Shrikumar and Ravi, 2007; Patwardhan and Mashelkar, 2009; Anand *et al.*, 2020; Banerjee *et al.*, 2021; Halder *et al.*, 2021). Dysentery was treated with Andrographolide from *Andrographis paniculata* (Burm.f.) Nees (Acanthaceae). Morphine was isolated from *Papaver somniferum* L. (Papaveraceae). *Mucuna pruriens* (L.) DC. (Fabaceae) served as a source of L-Dopa (Tandon *et al.*, 2021), a substitute for Dopamine and *Taxus brevifolia* Nutt. (Taxaceae) gave paclitaxel, an antineoplastic drug (Jarukamjorn and Nemoto, 2008; Katiyar *et al.*, 2012; Das *et al.*, 2021). Theasinensin D from *Camellia sinensis* (L.) Kuntze (= *Thea sinensis* L., Theaceae) can be used against human immunodeficiency virus (Hashimoto *et al.*, 1996; Fassina *et al.*, 2002; Nance and Shearer, 2003: Liu *et al.*, 2005; Bhutani and Gohil, 2010) and *Selaginella bryopteris* (L.) Baker (Selaginellaceae) is effective to kill *Plasmodium falciparum* (Kunert *et al.*, 2008; Bhutani and Gohil, 2010; Cao *et al.*, 2010; Pandey *et al.*, 2017;). Unani medicine is a popular choice among the urban population of Pakistan (Shaikh and Hatcher, 2005; Shaikh *et al.*, 2009). Twenty-nine medicinal plants like *Achyranthes aspera* L. (Amaranthaceae) for kidney stones, *Adiantum incisum* Forssk. (Pteridaceae) for bronchitis, *Aerva javanica* (Burm.f.) Juss. ex Schult. (Amaranthaceae) for flatulence and *Senna occidentalis* (L.) Link (= *Cassia occidentalis* L.) for snake bites, etc., are used in the urban population of Mirpur district (Mahmood *et al.*, 2011). Mianwali district inhabitants used 26 medicinal plants including *F. religiosa* L. (Moraceae) for menstrual disorders, *Morus alba* L. (Moraceae) for diabetes and hypertension, *Fagonia arabica* L. (Zygophyllaceae) for diabetes and *Morus nigra* L. (Moraceae) as a laxative (Qureshi *et al.*, 2007). In Abbottabad, 47 plant species like *Colchicum luteum* Baker (Colchicaceae), *Lactuca serriola* L. (Asteraceae) and *Cyperus rotundus* L. (Cyperaceae) are used for treating different ailments (Qureshi *et al.*, 2007). In Mastung district of Balochistan, 102 plants are used as herbal treatments out of which *Caralluma tuberculata*  N.E.Br. (Apocynaceae), *Citrullus colocynthis* (L.) Schrad. (Cucurbitaceae) and *Mentha longifolia* (L.) L*.* (Lamiaceae) were the most common species (Bibi *et al.*, 2014). Traditional Jamu medicine is widely used in Yogyakarta, Indonesia (Torri, 2013). In Dhaka, Bangladesh, 37 anti-diabetic plants are used such as *Coccinia grandis* (L.) Voigt (= *Coccinia indica* Wight & Arn., Cucurbitaceae), *A. indica* A. Juss. (Meliaceae), *T. chebula* Retz. (Combretaceae)*, Ficus racemosa* L. (Moraceae), *Momordica charantia* L. (Cucurbitaceae) and *Swietenia mahagoni* (L.) Jacq. (Meliaceae) (Ocvirk *et al.*, 2013). Usage of traditional Chinese medicine was seen in children in Singapore, again showing the cross-cultural manifestation of urban ethnobotanical knowledge (Loh, 2009).

### Urban ethnopharmacology in Australia

Australian urban people prefer using alternative herbal medicine along with conventional treatments for faster healing (Fisher *et al.*, 2018). Around 48% of the overall population in Australia uses alternative medicine, a vast number of whom were urban and mostly educated women (MacLennan *et al.*, 1996). CAM practisers are consulted by 28% urban women (Adams *et al.*, 2011). In a study in 2002, it was found that 32% insured urban adults treat neuromuscular diseases with alternative medicine in Washington State (Lind *et al.*, 2009). Some of these herbal medicines, like Ginseng, were self-prescribed (Steel *et al.*, 2018). Prevalence of use of Chinese medicine again shows the cross-cultural nature of TBK (Sibbritt *et al.*, 2013). According to a population survey conducted in the year 2007, in Victoria, 90% of the population believe medicinal plant usage is beneficial and 22.6% had used some herbs in their lifetime (Zhang *et al.*, 2008). Tea [*C. sinensis* (L.) Kuntze (= *Thea sinensis* L., Theaceae)], *A. vera* and garlic were the most popular. Ginseng, Peppermint, Chamomile, Ginkgo, Senna, Valerian, Dandelion, Liquorice and St John’s Wart were some of the other herbs identified (Zhang *et al.*, 2008). However, as herbal treatment is becoming increasingly popular among the urban people and is falling under health insurance policies, the cost of treatment is escalating. Hence, soon like in the USA, herbal medicine might be a luxury of the rich and privileged even in Australia.

## Yum Kaax: role of plants in religion

**Figure 4:**
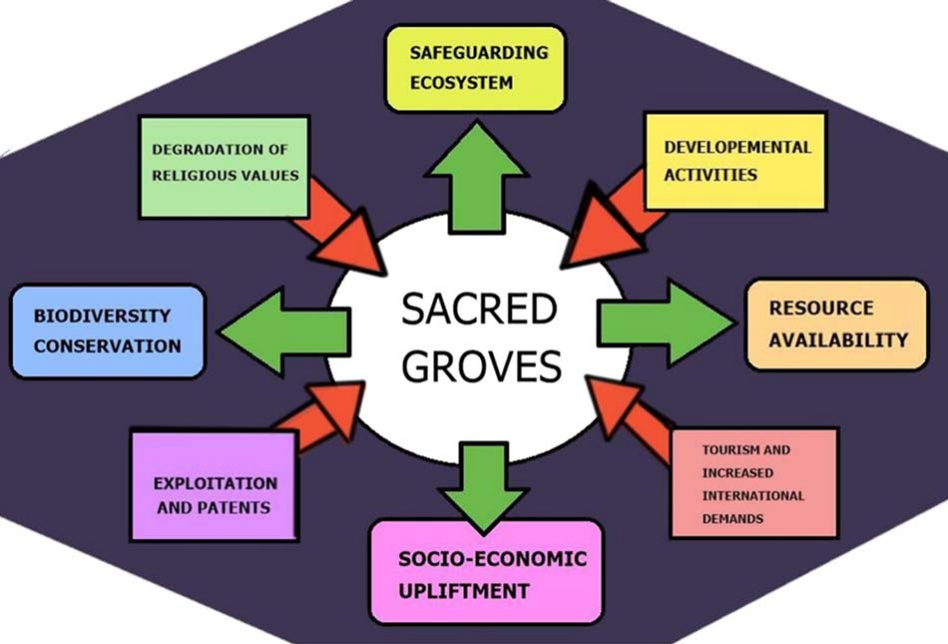
Role of sacred groves (green arrows) and factors responsible their degradation (red arrows).

**Figure 5:**
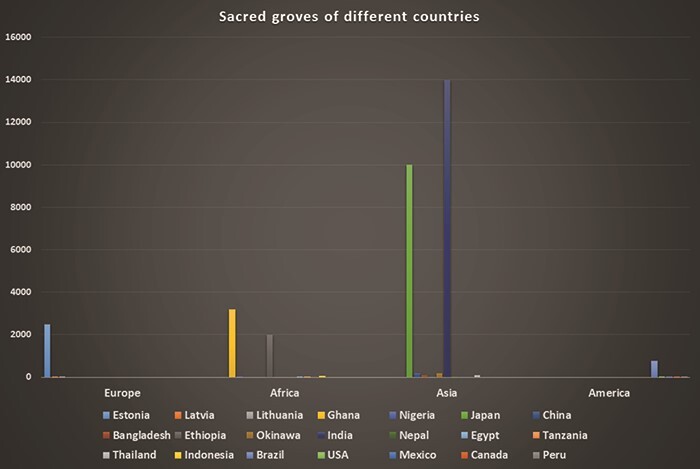
Sacred groves of different countries. Y-axis, number of sacred groves by country.

**Figure 6:**
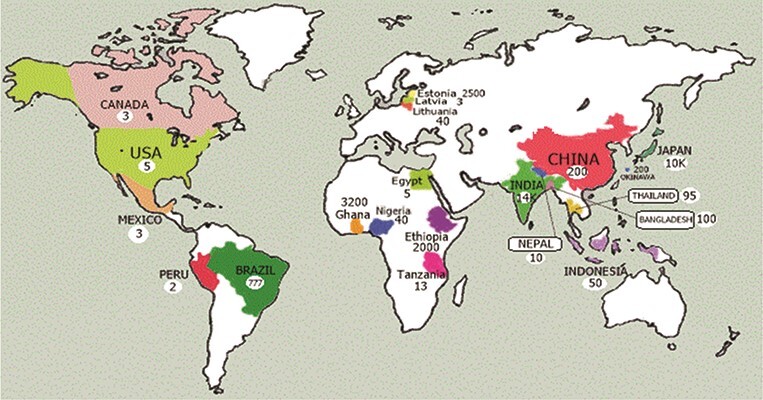
Map of sacred groves in the world.

Since ages, plants and their products have been used as offerings in religious ceremonies (Kessler *et al.*, 2013; Frazão-Moreira, 2016; Quiroz and van Andel, 2018) or have been worshipped as sacred deities, which play an important role in their conservation. Majority of these plants had medicinal values. The God-fearing nature of human beings obliged them to protect both domestic and wild varieties and hence these practices acted like gene banks (Child, 1993). From Adam and Eve’s expulsion from the Garden of Eden resulting from an apple to the tree of Bodhi, different plant species are deep rooted in almost every religion and mythology persisting on Earth. Human beings, who otherwise are selfish and have no empathy for nature, shift to sustainable use or cultivation of these special botanicals since they have to continue these rituals for ages. In some cases, religious leaders also play a substantial role in shaping the mind of a population towards conservation needs. Harming or cutting such trees is considered to be a sin and hence it is an effective tool to involve locals in safe guarding nature. These groups not only restrict their own consumption of the plants but also prevent overuse or theft by outsiders. Sacred groves are thickets or woodlands bearing plants of religious significance to certain communities or might be locations of important historical or mythological events (Adeniyi *et al.*, 2018; Mequanint *et al.*, 2020). The significance and the factors responsible for degradation of sacred groves have been depicted in Fig. 4. There are more than 100 000 sacred groves around the world, mainly concentrated in India, Nepal, South America, Japan and parts of Africa, some of which serving even as micro biosphere reserves. A graphical representation of sacred groves of the world and a map of country-wise occurrence major sacred groves are presented in Figs 5 and 6, respectively. The paganism and polytheistic religions teach nature worship and hence are mainly involved in the maintenance of these groves. As education and financial stability lead to the development and thus degradation of religious and cultural values, people tend to forget the important and sacredness of these groves leading to their exploitation and negligence of conservation.

### Plants in Christianity and Islam

For Monotheistic religions, i.e. the Abrahamic religions ‘Nature’ and hence trees ‘continue to be a source of Evil’. Satan came in the form of a snake enticing Eve to take a bite from the apple [*Malus domestica* Borkh. (Rosaceae)] of the tree of wisdom, which ultimately led to the Original Sin. In Genesis 1.29, it is written that God has instructed man, ‘See, I give you every seed-bearing plant that is upon all the earth, and every tree that has seed-bearing fruit; they shall be yours for food.’ The Bible has references to more than 36 trees (Coder, 2011; Evans, 2014) and is itself known as ‘The Tree of Life’ according to Proverbs3:18 Joyce Kilmer expressed ‘I think that I shall never see, a poem as lovely as a tree’, while studying Biblical verses mentioning trees. Old Testament predicted that Jesus’s death will occur on a tree and it was fulfilled, since the cross was made up of dogwood [*Cornus* sp., Cornaceae]. In John15:1, Jesus referred to himself as the ‘True Vine’ and his father as ‘The Gardener’. Trees are associated with a number of characters in Bible, such as Noah and the olive branch [*O. europaea* L. (Oleaceae)], Abraham rested under ‘the Oaks of Mamre’ [*Quercus* sp, Fagaceae], Zacchaeus ascended ‘The sycamore fig’ [*Ficus carica* L., Moraceae], Jacob and Almond trees [*Prunus dulcis* (Mill.) D.A. Webb, Rosaceae], Elijah and Juniper tree [*Juniperus* sp., Cupressaceae] and David with Balsam trees [*Abies balsamea* (L.) Mill., Pinaceae]. In Exodus, Deuteronomy and Kings, Bible associated paganism with sacred groves and ordered Jews to burn pagan groves. Supplementary Table 2 lists the trees mentioned in Biblical tales and verses.

**Table 1:** List of plants mentioned in Islamic religious texts

Common names	Scientific names	References
Apple	*M. domestica* Borkh.	Quran 2:7–286
Corn	*Zea mays* L.	Quran 55:5–72
Cucumber	*Cucumis sativus* L.	Quran 2:61
Date Palm	*Phoenix dactylifera* L.	Quran55:5–72
Fig	*F. carica* L.	Quran 95:1–8
Garlic	*A. sativum* L.	Quran 2:61
Gourd	*Lagenaria siceraria* (Molina) Standl.	al-Saaffaat 37:139, 142–146
Grape	*Vitis vinifera* L.	Quran 17:91
Honeyberry	*Celtis australis* L.	Quran 34:10–18
Lentil	*Lens culinaris* Medik. (= *Lens esculenta* Moench)	Quran 2:61
Olive	*O. europaea* L.	Quran 95:1–8
Onion	*A. cepa* L.	Quran 2:61
Palm tree	Arecaceae Bercht. & J. Presl	Quran 59:3
Pomegranate	*Punica granatum* L.	Quran 55:5–72
Tamarisk	*Tamarix aphylla* (L.) H.Karst.	Quran 34:10–18
Wheat	*Triticum* sp.	Quran 2:61

Islam, similar to Christianity, is another religion that believes in a single God and considers Nature to be opposite to God. There were many incidences in history where sacred groves were not protected by the Muslims (Campbell, 2005; Dafni, 2006). Many scholars also preached that trees can never be sacred (Dafni, 2011). In spite of all these prejudices, a number of trees have been mentioned in the Holy texts (Table 1), particularly the Sidra al-Munahā or the sacred lote tree denoting the edge of the 7th heaven, cypress, olive, date and fig trees. Graveyards have a lot of trees and these grounds are considered as holy and entry might be restricted (Dafni, 2006).

### Plants in Hinduism and Buddhism

Polytheistic religions value nature, hence Hinduism gives importance to considering trees as sacred beings. Nature worship is practiced as several trees are seen as Gods like Peepal, Banyan, banana, Ashoka and Neem. Majority of these plants have medicinal properties. Trees are worshipped as their products, help in healing and provide food and nourishment. A list of some of the species are described in Table 2. Trees like Tulsi are sometimes found in the majority of Hindu households since keeping them are as compulsory as keeping shrines. Several natural products are used in ritualistic practices or as offerings to deities. Apart from nature worship, several forests and groves are directly associated with mythological stories and hence entry is restricted and commercial overexploitation is forbidden. Hinduism also considers all beings as sentient and connected directly to Brahma, the creator (Framarin, 2014). They are also part of the ‘Cycle of Death and Rebirth’ (Hall, 2011) and are able to experience ‘pain and happiness’ (Dwivedi, 1990) and hence hurting them is considered to be unholy. There are also festivals where tree planting is a ritual and the trees are planted according to astrology (Coward, 2003; Haberman, 2013). Planting trees and conservation of groves are considered of high religious merit since groves and forests serve as religious prayer sites (Nath and Mukherjee, 2015; Sarma and Devi, 2015).

**Table 2:** List of plants of religious and medicinal significance in Hinduism

Common names	Scientific names	Local names
Wood Apple	*Aegle marmelos* (L.) Corrêa	Bel
Indian Wormwood	*Artemisia nilagirica* (C.B. Clarke) Pamp.	Kunju
Jackfruit	*Artocarpus heterophyllus* Lam.	Kathal
Indian Lilac	*A. indica* A. Juss.	Neem
Himalayan Birch	*Betula utilis* D. Don	Bhojpatra
Ngai Camphor	*Blumea balsamifera* (L.) DC.	Bari ilaichi
Rubber Bush	*Calotropis procera* (Aiton) Dryand.	Aak
Himalayan Cedar	*Cedrus deodara* (Roxb. ex D. Don) G. Don	Deodar
Camphor	*Cinnamomum camphora* (L.) J. Presl	Kapur
Bermuda grass	*Cynodon dactylon* (L.) Pers.	Dhoob
Jimson weed	*Datura stramonium* L.	Dhutra
Halfa grass	*Desmostachya bipinnata* (L.) Stapf	Kush
Woodenbegar	*Elaeocarpus serratus* L. (= *Elaeocarpus ganitrus* Roxb. ex G. Don)	Rudraksh
Indian Gooseberry	*P. emblica* L. (= *Emblica officinalis* Gaertn.)	Amla
Indian Coral Tree	*Erythrina variegata* L. (= *Erythrina indica* Lam.)	Paribhadraka
Banyan fig	*Ficus benghalensis* L.	Bargad
Sacred fig	*F. religiosa* L.	Pipal
Mango	*Mangifera indica* L.	Aam
Banana	*Musa* × *paradisiaca* L.	Kela
Basil	*Ocimum tenuiflorum* L. (= *Ocimum sanctum* L.)	Tulsi
Asian rice	*Oryza sativa* L.	Chawal
Long Leaf Indian Pine	*Pinus roxburghii* Sarg.	Chir
Betel Vine	*P. betle* L.	Paan
Himalayan Cherry	*Prinsepia utilis* Royle	Bhakel
Spunge Tree	*Prosopis cineraria* (L.) Druce	Khejri
Bird Cherry	*Prunus cerasoides* Buch.-Ham. ex D. Don	Paiya
Pomegranate	*P. granatum* L.	Anar
Woolly Oak	*Quercus oblongata* D.Don (= *Quercus leucotrichophora A. Camus*)	Banjh
Sandal	*S. album* L.	Chandan
Queen of the Night	*Saussurea obvallata* (DC.) Edgew.	Brahmakamal
White Marudah	*T. arjuna* (Roxb. ex DC.) Wight & Arn.	Arjun
Indian Mahogany	*Toona ciliata* M. Roem.	Tun
Indonesian lemon pepper	*Zanthoxylum acanthopodium* DC.	Timoor
Winged Prickly Ash	*Zanthoxylum armatum* DC.	Tejphal

Buddhism is neither monotheistic nor polytheistic, rather than giving all control and power to single or multiple gods, it gives importance to humanity, self-control and spirituality. Harming any living being including plants is considered sinful. Several plants are associated with the life of Buddha and are considered to be holy (Table 3), partly because Buddhism emerged from Hinduism and some of the principles have been retained (Hrynkow, 2017) and obtaining the rest of it from animism beliefs of Shinto religion (Omura, 2004). Ashoka tree is associated with Buddha’s birth and Peepal tree is the Bodhi tree. Furthermore, natural products are used as offerings and in other rituals by monks including some timber producing trees (Sahni, 2007; Upadhyay and Prasad, 2011).

**Table 3:** Plants of religious importance in Buddhism

Sl. No.	Common names	Scientific names
1	Acacia	*Acacia rugata* (Lam.) Fawc. & Rendle
2	Anatto tree	*Bixa orellana* L.
3	Ashoka Tree	*Saraca indica* L.
4	Ba jiao	*Musa* × *paradisiaca* L. (= *M. sapientum* L.)
5	Banana	*Musa nana* Lour.
6	Basil/ Tulasi	*O. basilicum* L.
7	Betel Leaf/ Paan	*P. betle* L. (= *P. betel* Blanco)
8	Bodhi Tree or Banyan	*F. religiosa* L.
9	Candle Nut tree	*Aleurites moluccanus* (L.) Willd.
10	Cannabis	*C. sativa* L.
11	China aster	*Chrysanthemum indicum* L.
12	Chittagong Wood	*Chukrasia tabularis* A. Juss.
13	Cinnamon	*Cinnamomum* sp.
14	Datura	*Datura stramonium* L.
15	English Beechwood/ Gamhar	*Gmelina arborea* Roxb.
16	Gardenia	*Gardenia sootepensis* Hutch.
17	Indian Gooseberry	*P. emblica* L.
18	Indian Mulberry	*Morinda officinalis* F.C. How
19	Ironwood	*Mesua ferrea* L.
20	Jackfruit	*A. heterophyllus* Lam.
21	Jasmine	*Jasminum* sp.
22	Juniper	*Juniperus* sp.
23	Lily	*Hedychium coronarium* J.Koenig (= *H. chrysoleucum* Hook.)
24	Lotus	*Nelumbo nucifera* Gaertn.
25	Magnolia/Champa	*Magnolia champaca* (L.) Baill. ex Pierre
26	Malay bushbeech	*G. arborea* Roxb.
27	Mango	*M. indica* L.
28	Margosa	*A. indica* A.Juss.
29	Oak Apple tree/ Bilva Tree	*A. marmelos* (L.) Corrêa
30	Paper Mulberry	*Broussonetia papyrifera* (L.) L’Hér. ex Vent.
31	Pine	*Pinus* sp.
32	Rhododendron	*Rhododendron* sp.
33	Rose Apple	*Syzygium sp.*
34	Saal tree	*Shorea robusta* Gaertn.
35	St. John’s Lily	*Crinum asiaticum* L.
36	Sweet potato	*Ipomoea batatas* (L.) Lam.
37	Tamarisk	*Tamarix* sp.
38	Teak	*Tectona grandis* L.f.
39	Water Lily	*Nymphaea* sp.
40	Wild Plum	*Z. jujuba* Mill.

## Discussion

While urban people have widely embraced TBK, there are still a number of stones in the direction of its advancement. Lack of scientific evidence validating the functionality and working principles of several of the organisms involved seems to be another drawback, since individuals struggle to believe in its fruitfulness. These prescriptions operate slower than conventional treatment and require cumbersome preparations, so customers opt for commercial pharmaceuticals that offer immediate or swift relief and are considerably simpler to use. Most individuals prefer to obtain their medicines at their doorsteps instead of wasting hours, pounding blends or extracting fluids from foliage, roots, etc. By educating the public, defining statistics and clarifying viewers’ notions, the media plays a vital role; but at the same time this could be exploited to propagate fake stories or generate fear among the public.

The terms Ayurveda and herbal are so common that simply claiming a product as herbal or adding these terms as a prefix improves the appeal of the product and increases its market demand. Many people miss the goods’ ingredient labels and businesses benefit from this. Therefore, the knowledge established by rural elders and sustained for generations have thus intensified capitalism. The privileged became wealthier and the scions of the original discoverers continued life in cages of deprivation (Verma, 2002). A plethora of biopiracy cases have occurred (De Werra, 2009; Dunagan, 2009; Efferth *et al.*, 2016; Ageh and Lall, 2019) where patents had been secured but the aboriginal populations concerned were not provided reasonable remunerations (Shiva, 2007; Shiva, 2016). Numerous American companies had copyrighted *A. indica* A. Juss. (Meliaceae) (Sheridan, 2005; Hamilton, 2006; Porter, 2006) and *Curcuma longa* L. (Zingiberaceae) (Udgaonkar, 2002; Schuler, 2004), two species that were used in India since ages. In Texas, where the company RiceTec declared Indian Basmati rice as ‘American-type Basmati’ and advertised it as its own invention, which was an example of both consumer misleading and biopiracy (Runguphan, 2004). This makes the potential use of these plant products illegal by members of the ethnic groups in that area, a phenomenon that directly challenges their livelihoods, totally beyond ethics and humanity. Scientists who patent these formulations justify their conduct by citing stronger benefits for the improvement of research and industrial applications such as drug discovery.

Nearly $5.4 trillion in royalties are tricked off every year from ethnic groups and tribes. Careful recording and maintenance by groups of these data or records could assist in preventing biopiracy, as in the case of turmeric in India. However, the best solution to this problem might be access to resources and advantage sharing. In short, it is the distribution of profits. Native tribes can patent their existing compositions and can then partner with corporations that, with the aid of branding and ambassadors, are involved in the production of raw materials and distribution of finished goods, but have to give a decent percentage of the revenue to the original inventors. In urban ethnopharmacology, IPR works as it gives exclusive rights, but the concept is reliant on collaborating. Bio-cultural legacy sees biodiversity and community as one and positions them in the category of collective rather than private ownership (Swiderska, 2006). The ethnic groups aid in the management and protection of natural resources with this approach. They are also able to avoid hunting, stealing and harm of resources by outsiders by making them guardians of the woods. However, they overexploit these tools as corporations get involved, contributing to their endangerment and rendering the ecological system vulnerable. This approach thus gives priority to preserving the whole and maintaining the balance of nature rather than individual plants.

Humans constitute an essential part of the planet. In the massive system called nature, all of us have respective roles. It is therefore our responsibility, as the most intelligent organisms, to manage and use these plant resources in sustainable ways for socioeconomic, cultural and economic growth. Thus, the line ‘country roads take me home’ may not necessarily be true since they often lead you towards new experiences and insights. Without human relationships, urban ethnopharmacology is nothing and thus if advanced nations persist with capitalizing developing countries, this might result in the disappearance of ancient medicinal and pastoral expertise. We should still realize that modernization needs the help of nature to suffice, but the reverse is contradictory.

## Conclusions

Medicinal plant usage by urban people was seen in all continents; however, the trends have been different. In the Americas, herbal medicines were either a part of healthcare of the poor in the developing countries or are been used as a health supplement by the rich in the developed countries, where the costs of these products are very high. Generally, in countries like Brazil, TBK was inversely proportional to education and financial status. In USA, alternative medicine could only be afforded by the rich and there was a general negative attitude towards the effectiveness of traditional plant medicines among the urban people. In Europe, small ethnic groups or immigrant groups living in metropolitan cities like London have kept the rich knowledge of botanical medicine alive. Urban people of South and East Asian countries like China, India, Pakistan, Bangladesh, Korea, Japan, Indonesia, etc., are highly dependent on herbal medicines belonging to all sections of the society, whereas negligible literature was found about usage in North and Western Asia. Herbal medicine industries in these countries generate substantial revenue. The trend in Africa is the same as southern Asia. Australian urban well-educated and employed women depend on alternative and herbal medicines, some of which are self-prescribed. However, as these treatments are being insured and becoming popular, the cost of treatment is increasing. Hence, soon like in the USA, herbal medicine might be a luxury of the rich and privileged even in Australia. In areas where tradition, culture and religion are an important part of the society, sacred groves and plants were prevalent, and some of them were of medicinal importance, since healing agents for diseases are considered holy. This plays a dominant role in their conservation. Monotheism normally shows plants in a negative light but does play some role in conservation by designating some groves as sacred or of ritualistic importance. Polytheism is inspired by animism and hence nature worship is frequently practiced. As a result, sacred groves are frequent in countries practicing polytheistic religions. However, there are still ways to go to recognize the contribution of the ethnic people with proper practices of bio-prospecting, avoiding biopiracy and protecting the consumer from misleading. Government, Non-Governmental Organizations and corporate sectors must carefully acknowledge the traditional wealth and the people and share a decent percentage of the revenue generated from commercial and sustainable utilization of ethnomedicinal knowledge.

## Supplementary material

Supplementary material is available at *Conservation Physiology* online.

## Author contributions

J.P. and A.D. conceptualized the theme and idea. T.D., U.A., S.C.S. and A.B.M. retrieved the literature and wrote the first draft of the manuscript. T.D. and U.A. designed the figures and tables. T.D., U.A. and D.A.P. arranged the references. U.A., D.A.P., R.K. and J.P. proofread and revised the manuscript. R.K., J.P. and A.D. critically read and made the final editing of the manuscript. S.C.S., J.P. and A.D. supervised. All authors reviewed the manuscript and agreed before submission.

## Conflict of Interest

The authors declare that this study (review) was conducted in the absence of any financial or commercial relationships that could be construed as a potential conflict of interest.

## Supplementary Material

Supplementary_Table_1_to_2_coab073
