# Editor’s Note on: Advancing urban ethnopharmacology: a modern concept of sustainability, conservation and cross-cultural adaptations of medicinal plant lore in the urban environment

**DOI:** 10.1093/conphys/coae049

**Published:** 2024-07-17

This is an Editor’s Note regarding: Tusheema Dutta, Uttpal Anand, Suchismita Chatterjee Saha, Abhijit Bhagwan Mane, Dorairaj Arvind Prasanth, Ramesh Kandimalla, Jarosław Proćków, Abhijit Dey, Advancingurban ethnopharmacology: a modern concept of sustainability, conservation and cross-cultural adaptations of medicinal plant lore in the urban environment, *Conservation Physiology*, Volume 9, Issue 1, 2021, coab073, https://doi.org/10.1093/conphys/coab073.

In July 2023, the Journal was contacted by a reader who raised various concerns around the accuracy of certain statements within the paper and missing citations. During the subsequent investigation, it was found that the original reviewers of the article failed to disclose that they had previously collaborated with the authors. The Journal obtained a post-publication review of the article, and this reviewer raised various concerns around a) the methodology described in the article and b) the robustness of the data provided to support the central hypothesis.

Following further discussion with the authors, the Editor-in-Chief feels that as this article is a narrative review article, as opposed to a systematic review, some of the conclusions may be subjective. The Editor wishes to acknowledge that other individuals writing a narrative review on the same topic may arrive at different conclusions than those presented herein.

During the investigation, the Journal received a further note of concern from Ben Gurion University of the Negev regarding an inappropriate affiliation to the institution by co-author Uttpal Anand. This affiliation has been corrected. Readers should also be aware that the Editor-in-Chief requested additional language edits for this article after acceptance, however these were not carried out due to a publisher error. As such, the article is not of the Journal’s usual standard in terms of clarity and correctness of the English.

