# Correction to: Advancing urban ethnopharmacology: a modern concept of sustainability, conservation and cross-cultural adaptations of medicinal plant lore in the urban environment

**DOI:** 10.1093/conphys/coae050

**Published:** 2024-07-18

This is a correction to: Tusheema Dutta, Uttpal Anand, Suchismita Chatterjee Saha, Abhijit Bhagwan Mane, Dorairaj Arvind Prasanth, Ramesh Kandimalla, Jarosław Proćków, Abhijit Dey, Advancing urban ethnopharmacology: a modern concept of sustainability, conservation and cross-cultural adaptations of medicinal plant lore in the urban environment, *Conservation Physiology*, Volume 9, Issue 1, 2021, coab073, https://doi.org/10.1093/conphys/coab073.

As published, this manuscript contained an error in author Uttpal Anand’s affiliation. This should read: Sam Higginbottom University of Agriculture, Technology and Sciences, Prayagraj, Uttar Pradesh, 211007, India.

These details have been corrected only in this correction notice to preserve the published version of record.

