# Supplementary material for: Advancing urban ethnopharmacology: a modern concept of sustainability, conservation and cross-cultural adaptations of medicinal plant lore in the urban environment
Source: Conserv Physiol. 2021 Sep 16;9(1):coab073. doi: 10.1093/conphys/coab073 (PMC8448427; doi:10.1093/conphys/coab073)
Supplement: Supplementary_Table_1_to_2_coab073 [file supplementary_table_1_to_2_coab073.docx]

**Supplementary Table 1.** Urban ethnobotanical reports from different parts of the globe

| **Family** | **Botanical names** | **State/ country** | **Ethnomedicinal use for/ against** | **Part used** | **Method of preparation** | **Route Of administration** | **Experimental setup** | **References** |
| --- | --- | --- | --- | --- | --- | --- | --- | --- |
| Acoraceae | *Acorus calamus* L. | Samogitia region, Lithuania, Europe | stomach pain, indigestion, gastric ulcer | roots | powder | one teaspoonful of powder taken with cold water twice daily | snowball technique and interview | Petkeviciute et al., 2010 |
|  |  |  | gastric distension, indigestion | roots | extract with alcohol | one teaspoonful once daily |  |  |
| Amaryllidaceae | *Allium cepa* L*.* | Samogitia region, Lithuania, Europe | cold | corm | juice | one teaspoonful of freshly pressed juice once daily | snowball technique and interview | Petkeviciute et al., 2010 |
|  |  |  | bronchitis | corm | decoction with honey | one cup in the evening |  |  |
| Acanthaceae | *Pachystachys lutea* Nees | Petrópolis, Rio de Janeiro, Brazil | pneumonia | roots | juice | oral | interview | Leitão et al., 2009 |
| Alismataceae | *Echinodorus grandiflorus* (Cham. & Schltdl.) Micheli | Petrópolis, Rio de Janeiro, Brazil | asthma | leaves | extract | oral | interview | Leitão et al., 2009 |
| Aizoaceae | *Zaleya pentandra* (L.) C. Jeffrey | Lahore, Pakistan | early abortion prevention | leaves,  roots | paste, juice | leaf and root juice are ingested | interview | Shah et al., 2016 |
| Asteraceae | *Baccharis elaeagnoides* Steud. ex Baker | Nova Friburgo, Rio de Janeiro, Brazil | gastric ulcer | leaves | extract | oral | participant observation, free listings, free and semi-structured interviews | Leitão et al., 2009 |
| Basellaceae | *Basella alba* L. | Rourkela, London, Lagos | constipation | leaves | juice | juice from leaves taken by pregnant woman and kids  to treat constipation | interviews | Kumar et al., 2018; Onuminya et al., 2018 |
| Brassicaceae | *Brassica glauca* (J.A.Schmidt) Kuntze | London | rheumatism and muscular pain | leaves  and  stem | paste | topical | interviews | Sandhu and Heinrich, 2005 |
| Cannabidaceae | *Cannabis sativa* L. | London,  Samogitia,  Siran,  French Guiana | sedative, analgesic, neural disorders, anti-arthritic | flower | paste and dried leaves | oral, pulmonary | interviews | Ahmad et al., 2009; Tareau et al., 2007; Petkeviciute et al., 2010 |
| Cucurbitaceae | *Cyclanthera pedata* (L.) Schrad. | Buenos Aires-La Plata, Río de la Plata region, Argentina | pharyngodynia, cardiac disorders, helminthiasis, gastropathy, bronchitis and  haemorrhoids | whole plant | powder | oral | participant observation, free listings, free and semi- structured interviews | Hurrell et al., 2015 |
| Euphorbiaceae | *Ricinus communis* L. | London | relieve swollen joints and tenders, dermatitis, nervous diseases, laxative, rheumatic pains | seed | seed oil | seeds and seed oil ingested and seed oil is applied to painful areas | interview | Sandhu and Heinrich, 2005 |
| Araceae | *Colocasia esculenta* (L.) Schott | Buenos Aires-La Plata, Río de la Plata region, Argentina | obsessive compulsive disorder | leaves | extract | topical | interview | Hurrell et al., 2015 |
| Asphodelaceae | *Aloe secundiflora* Engl. | Thika and Nairobi, Kenya, Africa | diabetes mellitus, cardiac disorder | stem bark | aqueous extract | oral | interview | Njoroge, 2012 |
|  | *Aloe vera* (L.) Burm.f. | London | minor burns, wound healing, sunburn, emollient | leaves | juice | topical | interview | Sandhu and Heinrich, 2005 |
| Asparagaceae | *Asparagus setaceus* (Kunth) Jessop | Thika and Nairobi, Kenya, Africa | diarrhoea | root | decoction | oral | field work, interview, preservation technique | Njoroge, 2012 |
| Asteraceae | *Artemisia japonica* Thunb. | Doda, Trans Himalayan region, Jammu & Kashmir, India | skin infections | whole plant | decoction | topical | field work, interview, preservation technique | Kaul et al., 1991 |
| Oxalidaceae | *Oxalis corniculata* L. | Doda, Trans Himalayan region, Jammu & Kashmir, India | dysentery | leaves | mixed with egg albumin | oral | field work, home remedies | Kaul et al., 1991 |
| Malvaceae | *Adansonia digitata* L. | Turin, Italy | burning lips | seed | powder | topical | field work, home remedies | Ellena et al., 2012 |
| Fabaceae | *Acacia seyal* Delile | Adeane, Casamance, Southern Senegal | toothache | leaves | paste | topical | field work, home remedies | Ellena et al., 2012 |
|  | *Senna alexandrina* Mill. (= *Cassia senna* L.) | London | stimulant and laxative | pods,  leaves | - | topical | interview | Sandhu and Heinrich, 2005 |
| Caricaceae | *Carica papaya* L. | Adeane, Casamance, Southern Senegal | anemia | leaves | decoction | oral | interview | Ellena et al., 2012 |
| Lamiaceae | *Plectranthus barbatus* Andrews (= *Coleus barbatus* (Andrews) Benth. ex G.Don) | Gamboa, Itacuruna Island, Sepetiba Bay, Rio de Janeiro, Brazil | liver problems | leaves | extract | oral | interview, data collection of medicinal plant, home remedies | Figueiredo et al., 1993 |
| Acoraceae | *Acorus calamus* L. | Western cape, South Africa | gastro-intestinal complaints | rhizomes | powder | oral | interview | Philander, 2011 |
| Anacardiaceae | *Mangifera indica* L. | Colombia, London | headache, laxative | fruits | juice | oral | interview | Ceuterick et al., 2008 |
| Apiaceae | *Cyclospermum leptophyllum* (Pers.) Sprague (= *Apium leptophyllum* (Pers.) F.Muell. ex Benth.) | Southern Ecuador, South America | diarrhoea, stomachaches | fruits | powder | oral | interview, historical sources, previous research paper | Bussmann, 2002 |
| Phyllanthaceae | *Phyllanthus niruri* L. | Peruvian Amazon, South America | liver and kidney problems | leaves | aqueous extract | oral | interview and questionnaires | Jernigan,  2012 |
| Rutaceae | *Citrus aurantiifolia* (Christm.) Swingle | Cabo Delgado, Mozambiqu, East Africa | cough and headaches | leaves, stem | extract | oral | interview, questionnaires | Matavele and Habib, 2000 |
| Solanaceae | *Capsicum annuum* L. (= *C. frutescens*  L.) | Bien Hoa,  Vietnam,  Hawai | diabetes mellitus | fruits | juice | oral | interview, printing photograph, questionnaires | Nguyen, 2003 |
| Asteraceae | *Cichorium intybus* L. | Pešter Plateau, Sandžak, South-Western  Serbia | good for  bones,  diarrhea | aerial  parts | tea | oral | interview,  questionnaires | Pieroni et al., 2011 |
| Amaranthaceae | *Beta vulgaris* L. | Dodruja  and  Greci,  Eastern  Romania | eye  inflammations | root | powder | topical | interview,  questionnaires | Pieroni et al.,  2012 |
| Lamiaceae | *Lavandula angustifolia* Mill. (= *L. spica* L.) | Seattle,  Western  Washing  ton | anxiety, skin  care | whole  plant | extract | oral | ethnographic  research,  interview | Poe et al., 2013 |
|  | *Mentha* × *piperita* L. | London,  Samogitia,  Buenos  Aires-La Plata, El Alto | nausea,  stomachache, flatulence, rheumatism,  neuralgia | leaves,  aerial  parts | juice,  infusion,  paste | oral, topical | interview | Sandhu and Heinrich, 2005 |
|  | *Leonotis nepetifolia* (L.) R.Br. | Petrópolis, Rio de Janeiro, Brazil | bronchial asthma, diarrhoea, fever, malaria and influenza | whole plant | paste | oral | interview | Leitão et al., 2009 |
| Asteraceae | *Achillea*  *millefolium* L. | Western Alps,  Piedmont,  NW Italy | headache,  bleeding  disorder | leaves | soups | oral | interview | Bellia and Pieroni, 2015 |
| Amaryllidaceae | *Allium*  *cepa* L. | London,  Afyonkara-  hisar  Samogtia | colds, flu and coughs analgesic  anti-inflammatory  anti-rheumatic  stomach ailments  aphrodisiac | bulbs | paste, soup,  infusion,  steam  inhalant,  juice, etc. | oral  ground and wrapped on  the wounded  area | interview,  questionna-  ires | Sandhu and Heinrich, 2005;  Ceuterick et al.,  2008; Kargıoğlu et al.,  2008 |
|  | *Allium*  *sativum* L. | London  Samogtia  Buenos Aires | bronchial infections  improving cardiac ability  (reducing cholesterol,  reducing blood pressure, etc) antidiabetic  antibiotic  insect bites | bulb,  corm | infusion with tea, paste,  alcohol  extraction, etc. | oral, external application | interview | Petkeviciute et al., 2010;  Sandhu and Heinrich, 2005  Ceuterick et al., 2008 |
|  | *Allium hookeri* Thwaites | Manipur urban  markets | lowers cholesterol and controls bp. | leaves,  roots | leaves are fried or chopped, roots  are fried | leaves and roots are fried and  eaten, chopped leaves are ingested | interview | Ayam, 2011 |
| Lauraceae | *Cinnamomum verum* J. Presl | London | vomiting, nausea,  colds and flu, diarrhoea,  insect bites | twigs,  bark | powder | topical | interview | Sandhu and Heinrich, 2005 |
| Iridaceae | *Crocus sativus* L. | Mecca, Duhok, L  London,  Thessaloniki | stimulant,  sedative,  abortifacient | stigma,  styles | dried stigma  and styles | oral | interview | Alqethami et al, 2017;  Mohammed and Akgül, 2018 |
| Hypericaceae | *Hypericum perforatum* L. | London | anti-depressant, anti-anxiety,  wound healing | flowering  tops | - | oral | interview | Sandhu and Heinrich, 2005 |
| Piperaceae | *Piper nigrum* L. | London | sore throat, dermatitis | fruit | dried unripe  fruit | oral, topical | interview | Sandhu and Heinrich, 2005 |
| Myristicaceae | *Myristica fragans* Houtt. | London | treats eczema, anti-rheumatic  gastroenteritis appetitizer | seeds | dried kernels | topical, oral | interview | Sandhu and Heinrich, 2005 |
| Zingiberaceae | *Zingiber officinale* Roscoe | London, Nova friburga,  Mecca,  Petropolis. | anti-emetic, soothes colds, cough and flu, indigestion, nausea sore throat | rhizome | paste,  decoction,  tea infusion | oral, topical | interview | Ceuterick et al., 2008; Leitão et al., 2009;  Alqethami et al, 2017 |
| Myrtaceae | *Myrtus communis* L. | London | antiseptic and helps in wound healing and ulcer treatment | leaves | essential oil | oil applied to wounds | interview | Sandhu and Heinrich, 2005 |
| Theaceae | *Thea sinensis* L. | London,  Mexico city,  French Guiana,  Ibadan,  Mauritius | analgesic, astringent,  anti-oxidant and relieves sore throat, | leaves,  buds | tea,  infusion | taken in the form of tea, i.e., boiled with water | semi-  structured interviews,  voucher  collection | Ceuterick et al., 2011; [Arenas](https://scholar.google.com/citations?user=qMAkOOkAAAAJ&hl=en&oi=sra) et al., 2013;  Mahomoodally, 2014; Ajayi and Moody, 2016 |
| Vitaceae | *Ampelocissus latifolia* (Roxb.) Planch | Nasik | ease delivery | root | raw,  infusion | roots are chewed or made into infusion ingested to ease delivery | ethnobotanical surveys,  field trips | Patil and Patil, 2005 |
| Moraceae | *Ficus racemosa* L. | Kolkata,  Mumbai,  Jodhpur,  Kathmandu. | dysmenorrhoea, dysentery,  dental problems, hair splitting, measles, diabetes | fruit,  leaves,  stem  bark | leaf juice,  raw fruits,  infusion of bark | leaf decoction is used for dysmenorrhoea, leaf juice for hair splitting, leaf latex for measles, bark infusion for dysentery and as mouth wash, fruits help controls diabetes | interviews,  ethnobotanical surveys,  field trips | Siwakoti and Siwakoti, 2000;  Malla, 1982  Das et al., 2015 |
| Poaceae | *Panicum miliaceum* L. | Nasik | laxative | grain | cooked grains | grains are cooked and ingested to ease digestion post pregnancy | ethnobotanical surveys,  field trips | Patil and Patil, 2005 |
| Malvaceae | *Grewia damine* Gaertn. | Lahore | anaemia, tumour | seeds,  leaves | - | seeds are used in anaemia. leaves are used to treat tumour | interviews | Shah et al., 2016 |
| Verbenaceae | *Lantana camara* L. | Lahore | carminative, diaphoretic,  antispasmodic | whole  plant | - | oral, topical | interviews | Shah et al., 2016 |
| Asphodelaceae | *Asphodelus tenuifolius* Cav. | Lahore | diuretic, ulcers | seeds | seed paste,  cooked seed | seeds are cooked and eaten to serve as diuretics and paste applied to ulcers | interviews | Shah et al., 2016 |

**Supplementary Table 2.** List of plants mentioned in the Bible

| **Sl. No.** | **Common names** | **Scientific names** | **References** |
| --- | --- | --- | --- |
| 1 | Abraham’s Bush | [*Vitex agnus-castus*](https://en.wikipedia.org/wiki/Vitex_agnus-castus) L. | Exodus 3:2 |
| 2 | Acacia | *Acacia tortilis* subsp. *raddiana* (Savi) Brenan (= [*Acacia raddiana*](https://en.wikipedia.org/wiki/Acacia_raddiana) Savi) | Exodus 25:10 |
| 3 | Agarwood | [*Aquilaria malaccensis*](https://en.wikipedia.org/wiki/Aquilaria_malaccensis) Lam. | Proverbs 7:17 |
| 4 | Almond | *Prunus dulcis* (Mill.) D.A. Webb | Genesis 43:11 |
| 5 | Aloe | [*Aloe succotrina*](https://en.wikipedia.org/wiki/Aloe_succotrina) Lam. | Proverbs 7:17 |
| 6 | Anemone | [*Anemone coronaria*](https://en.wikipedia.org/wiki/Anemone_coronaria) L. | Matthew 6:28 |
| 7 | Anise | [*Pimpinella anisum*](https://en.wikipedia.org/wiki/Pimpinella_anisum) L. | Matthew 23:23 |
| 8 | Apple | [*Malus domestica*](https://en.wikipedia.org/wiki/Malus_domestica) Borkh. | Genesis 2:7 and Job 31:39 |
| 9 | Athel tree (Tamarisk) | [*Tamarix aphylla*](https://en.wikipedia.org/wiki/Tamarix_aphylla) (L.) H. Karst. | Genesis 21:33 |
| 10 | Balsam | *Abies balsamea* (L.) Mill. | Exodus |
| 11 | Barley | *Hordeum* sp. | Numbers 5:15 |
| 12 | Bay | *Laurus nobilis* L. | Psalm 37:35 |
| 13 | Bdellium | *Commiphora africana* (A. Rich.) Engl. | Genesis 2:12; Numbers 11:7 |
| 14 | Bean | *Vicia faba* L. | Ezekiel 4:9 |
| 15 | Black Cumin | *Nigella sativa* L. | Isaiah 44:14 |
| 16 | Blackberry | *Rubus sanctus* Schreb. | Exodus 3:2 |
| 17 | Box | *Buxus sempervirens* L. | Isaiah 41:19 |
| 18 | Boxthorn | *Lycium europaeum* L. | Proverbs 22:5 |
| 19 | Bramble | *Rubus ulmifolius* Schott | Judges 9:15 |
| 20 | Broom | *Calycotome villosa* (Poir.) Link | Psalm 120:4, 1 Kings 19:4 |
| 21 | Bulrush | *Typha* sp. | Exodus 2:3 |
| 22 | Caper | *Capparis spinosa* L. | Ecclesiastes 12:5 |
| 23 | Carob | *Ceratonia siliqua* L. | Luke 15:16, Matthew 3:1 |
| 24 | Cassia | *Cinnamomum iners* Reinw. ex Blume | Exodus 30:24, Psalm 45:8, Job 42:14 |
| 25 | Castor | *Ricinus communis* L. | Jonah 4:9 |
| 26 | Cedar of Lebanon | *Cedrus libani* A. Rich. | 1 Kings 5:10, 2 Kings 19:23 |
| 27 | Cinnamon | *Cinnamomum verum* J.Presl (= *Cinnamomum zeylanicum* Blume) | Proverbs 7:17 |
| 28 | Citron | *Citrus medica* L. | Jeremiah 15:9 |
| 29 | Cockle | *Agrostemma githago* L. | Job 31:40 |
| 30 | Coriander | *Coriandrum sativum* L. | Exodus 16:31 |
| 31 | Cotton | *Gossypium herbaceum* L. | Esther 1:6 |
| 32 | Crocus | *Crocus sativus* L. | Song of Solomon 2:1, Isaiah 35:1 |
| 33 | Cucumber | *Cucumis melo* (Mill.) J.H. Kirkbr. | Numbers 11:5 |
| 34 | Cumin | *Cuminum cyminum* L. | Isaiah 28:27 |
| 35 | Cypress | *Cupressus sempervirens* L. | Isaiah 44:14 |
| 36 | Darnel | *Lolium temulentum* L. | Matthew 13:25–40 |
| 37 | Date Palm | *Phoenix sylvestris* (L.) Roxb. (= *Elate sylvestris* L.) | Song of Solomon 5:11; 7:7, 8, John 12:13 |
| 38 | Dill | *Anethum graveolens* L. | Matthew 23:23 |
| 39 | Dove’s Dung | *Ornithogalum narbonense* L. | 2 Kings 6:25 |
| 40 | Elm | *Ulmus minor* subsp. *canescens* (Melville) Browicz & Ziel. | Song of Solomon 5:11; 7:7, 8, John 12:13 |
| 41 | Fig | *Ficus carica* L. | Joel 1:7 |
| 42 | Fir | *Abies cilicica* (Antoine & Kotschy) Carrière | 1 Kings |
| 43 | Flax | *Linum usitatissimum* L. | Proverbs 31:13 |
| 44 | Frankincense | *Boswellia thurifera* Roxb. ex Fleming | Matthew 2:10, 11 |
| 45 | Garlic | *Allium sativum* L. | Numbers 11:5 |
| 46 | Grape | *Vitis vinifera* L. | Genesis 40:10 |
| 47 | Hedge of thorns | *Solanum incanum* L. | Proverbs 15:19 |
| 48 | Hemlock | *Conium maculatum* L. | Amos 6:12 |
| 49 | Henna | *Lawsonia inermis* L. | Song of Solomon 1:14 |
| 50 | Hyssop | *Hyssopus officinalis* L. | Leviticus 14:52 |
| 51 | Iris | *Iris palaestina* (Baker) Boiss. | 1 Kings 7:22 |
| 52 | Judas tree | *Cercis siliquastrum* L. | Matthew 27:5 |
| 53 | Jujube | *Ziziphus spina-christi* (L.) Desf. | Matthew 27:29 |
| 54 | Juniper | *Juniperus excelsa* M. Bieb. | Jeremiah 17:6, 48:6 |
| 55 | Leek | *Allium ampeloprasum* L. (= *Allium porrum* L.) | Numbers 11:5 |
| 56 | Lentil | *Lens culinaris* Medik. (= [*Lens esculenta*](https://en.wikipedia.org/wiki/Lens_esculenta) Moench) | 2 Samuel 17:28 |
| 57 | Lily of Valley | *Convallaria majalis* L. | Song 2:1 |
| 58 | Linen | *Linum usitatissimum* L. | Exodus 35:25 |
| 59 | Lotus | *Nelumbo nucifera* Gaertn. | Job 40:21–22 |
| 60 | Mallow | *Atriplex halimus* L. | Job 30:4 |
| 61 | Mandrake | *Mandragora autumnalis* Bertol. | Genesis 30:15 |
| 62 | Manna | *Alhagi maurorum* Medik. (= *Alhagi camelorum* DC.) | Numbers 11:7 |
| 63 | Mint | *Mentha* sp. | Matthew 23:23 |
| 64 | Mulberry | *Morus* sp. | Luke 17:6 |
| 65 | Mustard | *Brassica nigra* (L.) K. Koch | Matthew 13:31 |
| 66 | Myrhh | *Commiphora guidottii* Chiov. ex Guid. | Genesis 37:25, 43:11 |
| 67 | Myrtle | *Myrtus communis* L. | Isaiah 55:33 |
| 68 | Nettle | *Acanthus spinosus* L. | Isaiah 55:33 |
| 69 | Oak | *Quercus coccifera* L. (= *Quercus calliprinos* Webb) | Joshua 24:26 |
| 70 | Olive | *Olea europaea* L. | Judges 9:9 |
| 71 | Onion | *Allium cepa* L. | Numbers 11:5 |
| 72 | Papyrus | *Cyperus papyrus* L. | Exodus 2:3, Job 40:21 |
| 73 | Pistachio | *Pistacia vera* L. | Song of Solomon 6:11 |
| 74 | Plane | *Platanus orientalis* L. | Ezekiel 31:8, Genesis 30:37 |
| 75 | Pomegranate | *Punica granatum* L. | Song of Solomon 7:12 |
| 76 | Poplar tree | *Populus* sp. | Isaiah 44:4 |
| 77 | Pursiane | *Portulaca oleracea* L. | Job 6:6 |
| 78 | Red Sandalwood | *Pterocarpus santalinus* L.f. | Chronicles 2:8; 9:10, 11 |
| 79 | Reed | *Phragmites* sp. | Ezekiel 40:vv., Job 40:21 |
| 80 | Rue | *Ruta graveolens* L. | Luke 11:42 |
| 81 | Rush | *Juncus* sp. | Isaiah 9:14 |
| 82 | Saffron | *Crocus* sp. | Song of Solomon 4:14 |
| 83 | Spelt | *Triticum spelta* L. | Isaiah 28:25 |
| 84 | Spikenard | *Nardostachys jatamansi* (D. Don) DC. | Song of Solomon 4:14 |
| 85 | Stacte | *Styrax officinalis* L. | Exodus 30:34 |
| 86 | Storax | *Liquidambar orientalis* Mill. | Exodus 30:34 |
| 87 | Terebinth | *Pistacia palaestina* Boiss. | Samuel 18:9 |
| 88 | Thistle | *Cardueae* sp. | Job 31:40, 2 Chronicles 25:18 |
| 89 | Tulip | *Tulipa agenensis* DC. | Song of Solomon 2:1 |
| 90 | Walnut | *Juglans regia* L. | Song of Solomon 6:11, Genesis 43:11 |
| 91 | Watermelon | *Citrullus lanatus* (Thunb.) Matsum. & Nakai | Numbers 11:5 |
| 92 | Wheat | *Triticum* sp. | Ezra 7:22 |
| 93 | White Sandalwood | *Santalum album* L. | Kings 10:11, 12 |
| 94 | Willow | *Salix alba* L. | Job 40:22 |
| 95 | Wormwood | *Artemisia absinthium* L. | Revelation 8:11 |
